# Evaluation of PEETER V1.0 urine sensors for measuring individual urination behavior of dairy cows

**DOI:** 10.3168/jdsc.2020-0019

**Published:** 2020-10-29

**Authors:** C.J. Marshall, M.R. Beck, K. Garrett, N. Beale, P. Gregorini

**Affiliations:** Faculty of Agriculture and Life Sciences, PO Box 85084, Lincoln University, Lincoln 7647, Christchurch, New Zealand

## Abstract

Due to environmental concerns around N leaching and NO_2_ emissions from intensive pastoral dairying systems, there has been an increase in research focused on mitigation strategies and on-animal technologies to evaluate urination behavior of grazing dairy cows. Nitrogen leaching and NO_2_ emissions are associated with urine nitrogen loading onto pasture, which is a function of urine nitrogen concentration and urine volume per urination event. The PEETER V1.0 urine sensor (Lincoln University, Christchurch, New Zealand) is a promising on-animal measurement technology; however, it has yet to be validated in vivo. The objective of this work was to validate the PEETER V1.0 urine sensor's estimations of individual urination events (i.e., urine volume). We fitted 15 Holstein-Friesian × Jersey lactating dairy cows (506 ± 35 kg of live weight, body condition score of 3.75 ± 0.25, and 150.4 ± 20.7 d in milk) with individual PEETER V1.0 sensors and placed them in metabolism crates for 72 h. Every urination event (n = 480) was collected manually and compared with the urine volume estimated by the PEETER V1.0 sensor to determine precision and accuracy using Lin's concordance correlation coefficient (CCC). The CCC is calculated as a function of the Pearson's correlation (precision) and bias correction factor (C_b_; C_b_ = 1 is perfect), and it demonstrates how far the values of the 2 methods are from perfect agreement (accuracy; i.e., a 45° line). The mean urination event volume (mean ± standard deviation) was 2.7 ± 0.94 and 2.6 ± 0.92 L for the actual and PEETER V1.0 sensor, respectively. The PEETER V1.0 sensor showed excellent precision (r = 0.90) with near-perfect accuracy (C_b_ = 1.00), and the CCC value was high (CCC = 0.90), indicating excellent agreement. Based on these results, the PEETER V1.0 urine sensor provides estimates that are precise and accurate. We conclude that the PEETER V1.0 sensor can be used to evaluate urination behavior of grazing dairy cows.

## Highlights

• PEETER V1.0 sensors are a simple and inexpensive way to monitor cow urination behavior
• PEETER V1.0 sensors were found to have excellent accuracy
• PEETER V1.0 sensors were shown to have excellent precision

## Graphical Abstract

**Figure:**
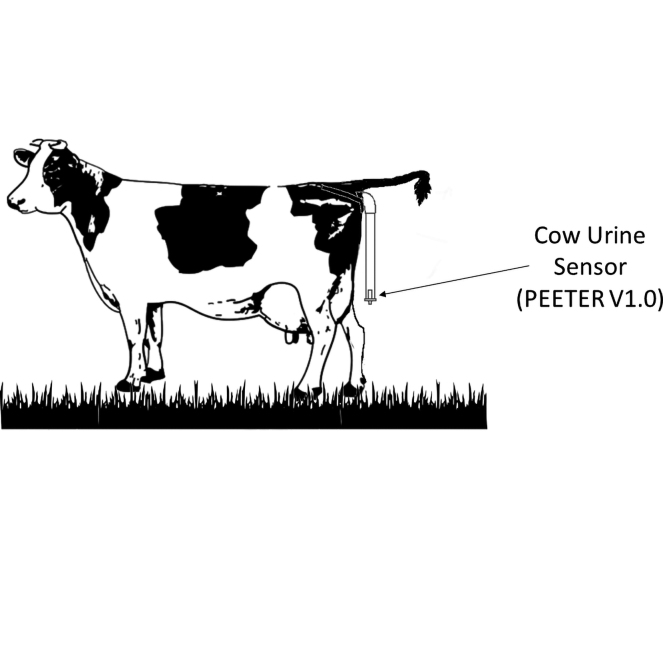
**Summary:** Increasing environmental concerns from intensive dairy production systems surrounding nitrogen loses has stimulated research and development on mitigation and monitoring technologies focusing on urination behavior of dairy cows. The Lincoln University PEETER V1.0 sensor is a promising piece of technology for monitoring cow urination behavior. The objective of this study was to validate the use of the PEETER V1.0 sensor in vivo. Fifteen lactating Holstein-Friesian × Jersey dairy cows were fitted with the PEETER V1.0 sensor and housed within metabolism crates for 72 h. Every urination event during this period was collected manually and compared with the PEETER V1.0 sensor estimations of each event. The PEETER V1.0 sensor was found to have both excellent precision and accuracy for estimating urination events and is therefore a promising piece of new technology for monitoring animal urination behavior.

Cattle urine characteristics, such as volume and nitrogen (N) concentration, play a large role in determining the levels of N deposited onto pastures and subsequently lost from the system as environmental pollutants. Urine patches are areas of high nitrate leaching due to the saturation of the swards and the soil's ability to utilize all of the N (Li et al., 2012). High N loading rates from urine patches are associated with negative environmental impacts (Marshall et al., 2020). Information regarding urination behavior such as volume per event and diurnal patterns will have large implications on grazing management, in terms of animal movements away from ecologically sensitive areas to areas where urine can be captured. A gap in research exists on urination behavior because of the logistics of measuring urination behavior in a field setting (Gregorini et al., 2018). The Lincoln University PEETER V1.0 (PEE meter; Lincoln University, Christchurch, New Zealand) sensor is a recent development that represent a lightweight and cost-effective solution for obtaining urination behavior data in the field. The sensor has been validated within a laboratory setting and used in several outdoor grazing trials (Mangwe et al., 2019, 2020; Marshall et al., 2020). However, PEETER sensor validation within a controlled environment using animals has yet to be conducted. Although laboratory validation has occurred, the laboratory represents a perfect environment and cannot account for variability caused by environmental and animal factors. Therefore, the ability for the sensor to create accurate and precise readings from an animal has not been tested. This illustrates the need for in vivo validation of the PEETER V1.0 sensor. As such, the objective of this study was to compare the accuracy and precision of the PEETER V1.0 sensor against manual measurements of urination events for volume per urination. We hypothesized that the PEETER V1.0 urine sensor will provide an accurate and precise method to measure urine behavioral characteristics in vivo.

All animal manipulations in the present study were conducted with approval of the Lincoln University Animal Ethics Committee (AEC 2019-25).

Fifteen Holstein-Friesian × Jersey lactating dairy cows (506 ± 35 kg of live weight, 3.75 ± 0.25 BCS, and 150.4 ± 20.7 DIM) were placed in metabolism crates for 72 h; animals had the ability to lie down within the metabolism crates. Animals were provided with ad libitum access to fresh water and were fed fresh-cut perennial ryegrass (*Lolium perenne* L.) and plantain (*Plantago lanceolata* L.) herbage allocated twice daily (0800 and 1600 h) and had an average intake of 16 ± 3 kg of DM/d. Herbage was harvested daily from the sward (with a surface sward height of 30 cm) at a height of 3 cm above the ground using a Haldrup (Haldrup GmbH, Ilshofen, Germany) harvester, stored in a 4°C chiller, and fed as unchopped fresh-cut herbage.

Four PEETER V1.0 urine sensors were provided by the PEETER development team and fit to each cow. The sensors consist of a Feather MO proto (Adafruit P2772; Adafruit Industries, New York, NY) microprocessor with an Adalogger Featherwing (Adafruit P2922; Adafruit Industries,) for memory and time keeping. A Honeywell 1PSI Diff 3.3v HSCMRRN001PDAA3 (Digi-Key 800 344-4539; Digi-Key Electronics, Minneapolis, MN) pressure sensor and a LiPo 800mAh battery with an approximate operating time of 48 h. The components are encased within a watertight acetal plastic tube with a total weight of 225 g (Mangwe et al., 2019). The sensor works based on the principle of differential pressure from the urine acting on the pressure-sensor inlet orifice. Volume (*V*) is calculated using a modified Bernoulli's equation, *V* = *bx*^EXP^, where *b* is CV [CV = 2gH (g = acceleration of gravity and H = height derived from flow time)] and *x* = Pascal's reading followed by the resulting extrapolation (Mangwe et al., 2019).

A harness was constructed using a vinyl fabric (Zephyr Vinyl; Spotlight, Australia) and a 3-dimensional (3D) printed mold made of acrylonitrile butadiene styrene printed using a UP-Mini 3D Printer (3D Printing Systems, Auckland, NZ) with a plastic sleeve to which the sensor attaches at the bottom via a locking mechanism secured with zip ties and glue. The harness is attached to the animal over the vulva using an approved biocompatible glue (we used Loctite 454; Henkel, Düsseldorf, Germany). The total weight of the harness and sensor together was ~400 g.

Animals were monitored by trained technicians for 72 h while housed in metabolism crates. Every urination event was collected by a technician by placing a bucket underneath the PEETER V1.0 sensor when an event occurred. The total weight of the event and the time of the event were then recorded by the technician to compare against the sensor's recording. A total collection tray was in place underneath the metabolism crate to capture any urine that may have been spilled or missed by the technicians. The tray was emptied every 12 h and the total urine volume recorded. An adjusted volume by urination event was calculated by dividing the total amount of urine in the tray after 12 h by the number of events counted in that time. We assumed that a constant amount of urine was lost per event; from this assumption, we calculated an adjusted urination weight per event to account for any urine in the total collection tray during each 12-h period.

A regression model was fit to the paired data using the “lm” function of base R (v.3.6.3; https://www.r-project.org/), with actual measured urine volume as the independent variable and sensor-estimated urine volumes as the dependent variable. The Pearson correlation coefficient was also determined between the 2 methods using the “cor.test” function of base R (v.3.6.3). To compare the validity of the urine sensors to measure the urine volume of individual urination events, we used Lin's concordance correlation coefficient (**CCC**; Lin, 1989, 2000). This technique combines precision and accuracy by determining how far the regression line of an alternative method and a “gold-standard” method is from the 45° line, which would indicate perfect agreement. This method calculates the CCC by multiplying the Pearson correlation coefficient (i.e., measure of precision) by a bias correction factor (**C_b_**), where a C_b_ of 1 indicates no deviation from the 45° line. Similar to the Pearson correlation, the CCC ranges from −1 to 1; however, only values near 1 indicate that the alternative method provides adequate estimates compared with the gold-standard measurement. There is typically no set definition for what ranges of CCC indicate no agreement, slight, fair, moderate, good, and excellent agreement but we defined these as <0, 0–0.20, 0.21–0.40, 0.41–0.60, 0.61–0.80, and >0.80, respectively. Lin (1989) also describes what is termed a scale shift (βnu;), which is the ratio of the method's standard deviation (**SD**), such that βnu; = 1 indicates that the methods have the same SD, and a location shift (*u*), which is the difference between the averages relative to the scale (i.e., analogous to the bias). The C_b_ is calculated using the *u* and βnu; values. All analysis of Lin's CCC was conducted using the “CCC” function of the DescTools package (Signorell et al., 2020). Graphics were generated using the “ggplot2” package (Wickham, 2016). All analysis was conducted using R software (v.3.6.3).

Figure 1 presents the regression line between actual measured urine volume (independent variable) and volume recorded by the PEETER V1.0 sensor (dependent variable). The regression analysis determined an intercept of 0.2 (SEM = 0.06) with a slope of 0.9 (SEM = 0.02).

**Figure 1:**
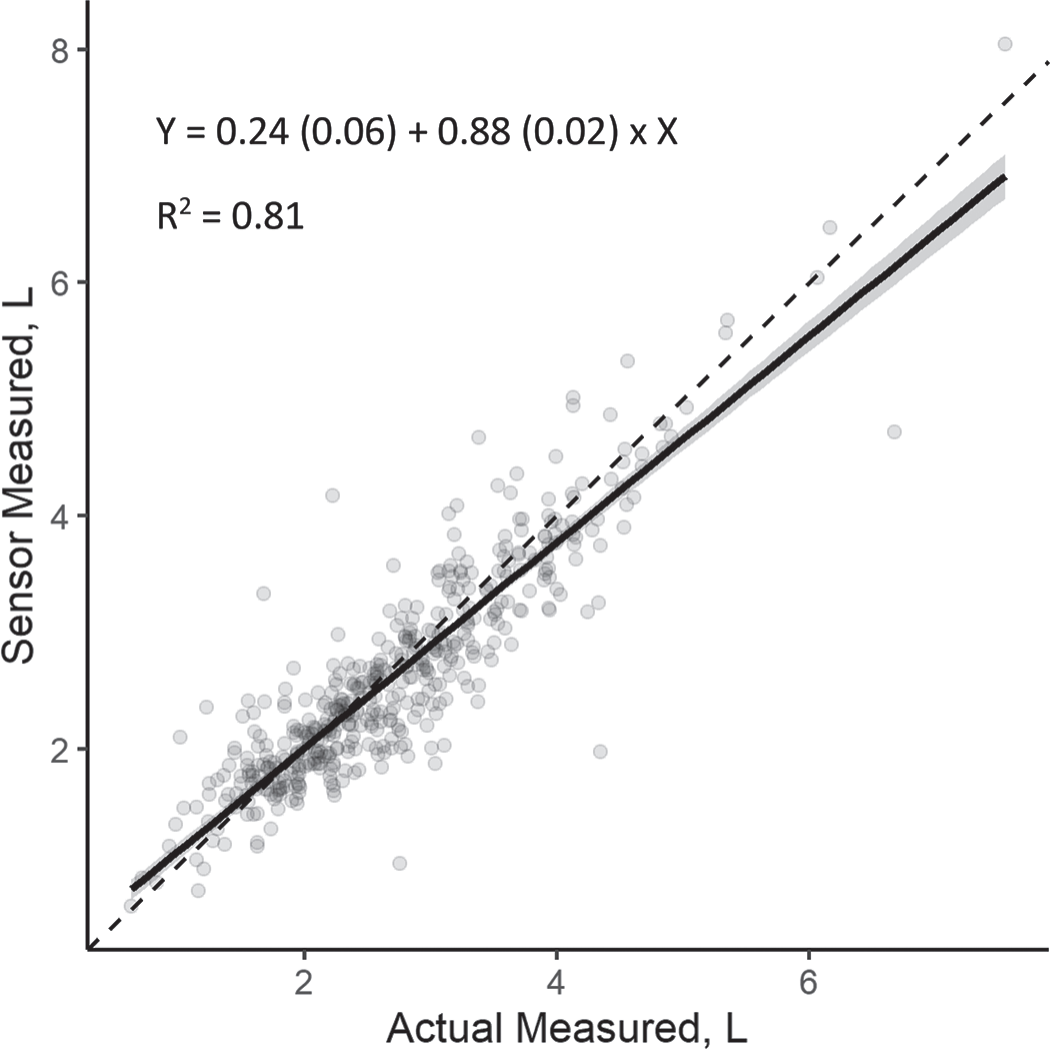
The regression line comparing actually measured urine volumes to urine volumes measured using the PEETER V1.0 urine sensor (sensor measured; Lincoln University, Christchurch, New Zealand). The solid line is the regression line, with the 95% CI shown by the shaded band. The dashed line is the 45° line. Each data point represents one paired urination event.

Table 1 presents descriptive statistics of actual urine volume measurements and PEETER V1.0 urine volume estimates. Numerically, there was little difference between the mean volume, SD, or minimum value (0.01 L, 0.02 L, and 0.02 L, respectively); however, a larger numerical difference was observed between the maximum values of 0.5 L, with the PEETER V1.0 sensor overestimating the maximum value.

**Table 1:** Descriptive and comparative statistics of actually measured urine volumes and urine volumes determined by the PEETER V1.0 urine sensors

Statistic	Actual	Sensor
Descriptive		
n	480	480
Mean	2.7	2.6
SD	0.94	0.92
Maximum value	7.55	8.05
Minimum value	0.63	0.65
Comparative^1^		
r	0.90	
C_b_	1.00	
CCC	0.90	
*u*	0.07	
ν	1.02	

1 r = Pearson's correlation coefficient; C_b_ = bias correction factor; CCC = Lin's concordance correlation coefficient, calculated as r × C_b_; *u* = location shift; that is, the mean bias; ν = scale shift, a measure of the difference in SD between the 2 methods (i.e., ν of 1 indicates that the methods have the same SD).

Table 1 also presents comparative statistics between the actual measured events compared with sensor- measured events. Both *u* and βnu; can be used as measures of accuracy between the 2 techniques. The *u* of 0.07 indicated a very low mean bias, with a difference of only 0.07 L between the average amounts reported by each technique. A βnu; of 1.02 indicates a small ratio in the standard deviations between the techniques, where a βnu; of 1 would indicate identical standard deviations. Both *u* and βnu; indicate good accuracy between the 2 techniques. A bias correction factor of 1 indicates that the best-fit line deviated negligibly from the 45° line, indicating a high degree of overall accuracy from the techniques. Pearson correlation can be used to evaluate the precision of the 2 techniques. The r-value of 0.90 indicated a strong positive linear relationship and a high level of precision from the 2 techniques. The CCC of 0.90 indicated a high level of agreement between the 2 techniques, representing an excellent level of accuracy and precision for the PEETER V1.0 sensor.

The objective of this study was to compare the accuracy and precision of the Lincoln University PEETER V1.0 sensor against actual measurements of urination events for quantity per event. Our hypothesis was that the PEETER V1.0 urine sensor would provide accurate and precise measurements of urine behavioral characteristics in vivo. The results of this study confirm our hypothesis and present strong evidence to support and confirm an excellent level of agreement between the actual measured urination events from dairy cows and the events recorded by the PEETER V1.0 sensor.

There was an overall tendency for the PEETER V1.0 sensor to underestimate the total amount urinated, especially at higher volumes, as seen in Figure 1. The likely reason for such underestimation could be the “overflow” mechanism built into the urine harness. There are vents incorporated at the top of the 3D-printed component of the harness, where urine can overflow if the sleeve of the harness becomes full of urine either because of a blockage or because the volume of urine is greater than the sleeve can hold. This was observed with larger urination events (events >5 L); in these scenarios, the overflowing urine would have been collected in the bucket that was placed underneath the sensor but not all of this urine would have passed through the sensor, resulting in a lower sensor recording compared with the physical measurement. The occurrence of large urination events resulting in urine overflow was low. Only 3% of events recorded in this study had a volume greater than 5 L, with an average overall urine volume of 2.9 L per event throughout the study. Several other studies in both grazing and indoor situations report a similar average urination volume per event of 2.9 to 3.0 L (Betteridge et al., 2013; Mangwe et al., 2019; Marshall et al., 2020), which is below the observed threshold for overflow with this current design. Additionally, a potential measurement error exists with the actual measured urine volumes due to human error, where any delay in the technician capturing urine from the harness at the start of the event will result in a deviation between the sensor and actual volume measured. We tried to control for this by creating an adjusted volume per event by calculating how much urine was captured in the total collection tray every 12 h and counting how many events occurred during this time. A constant rate of missed urine was assumed, but it is impossible to tell how much was missed per event, which may explain some amount of the variation seen between the 2 techniques. It is unlikely that any additional variability would be detected in results as a result of actual urination events being larger than calibration events (5 L) because the PEETER V1.0 sensor measures volume based on pressure as flowrate and time. If the incoming volume flow rate is less than the sensor flowrate maximum plus sleeve volume containment, the sensor will remain accurate. The main constraint to accurate measurements at higher volumes is urine leaving from the overflow vent because this is beyond the sensor's range. This could be addressed by either having a larger collection tube attached to the animal, which presents other problems such as being too long and annoying the animal, or increasing the flowrate, which potentially lowers the sensor sensitivity. Both issues are being addressed in the development program of the next version of the sensor, as is a proportional sample bottle to allow for chemical analysis of the urine.

The onboard clock used by the PEETER V1.0 sensor had several recordings with incoherent dates and times. If these sensors were used in a field setting without any reference point of when the first urination event occurred, it would be impossible for researchers to create an accurate diurnal pattern of urination. It is recommended, therefore, that the onboard clock be calibrated per sensor before the start of any experimentation; stimulating the animal to urinate at a known time point could also provide a known reference point for researchers to account for any deviations in timekeeping from the PEETER V1.0 sensor.

The battery life of the sensors was also found to be unsatisfactory, with many sensors having a much shorter lifespan than expected. This was due to the software running on the sensor and its large power consumption. This short battery life precludes these sensors from use in any experiment where the experimenters do not have regular access to the sensors to check battery life. In the present study, the animals were constantly observed, so batteries could be replaced regularly to minimize loss of data. Battery life concerns have been addressed in the latest version of the sensor, with a change in software resulting in 120-h run times. This will allow for the sensor to be attached to animals for longer periods and increase the sensor's usability in outdoor settings.

The addition of an onboard global positioning system (GPS) or other location device would be valuable for future research on the urination behavior of cattle. Having knowledge of where and when cattle are urinating would allow for further fine-tuning of management systems and modeling of urination behavior and therefore nitrate leaching hotspots. Laboratory validation before and after the use of the PEETER V1.0 sensors is recommended to determine any misalignment or defects that could produce skewed results during the experiment. In this way, it may be possible to calibrate the results if they are shown to be consistently inaccurate during calibration.

The Lincoln University PEETER V1.0 sensor appears to be an excellent tool to study urination behavior of cattle because it provides an excellent estimation of urine volume per event. Calibration before and after use is recommended to account for any deviations in results that may occur per sensor. Detailed descriptions of urination behavior help in the development of management solutions to reduce these environmental impacts and potentially pave the way toward selective breeding based on urination behavior.
